# A deletion affecting an LRR-RLK gene co-segregates with the fruit flat shape trait in peach

**DOI:** 10.1038/s41598-017-07022-0

**Published:** 2017-07-27

**Authors:** Elena López-Girona, Yu Zhang, Iban Eduardo, José Ramón Hernández Mora, Konstantinos G. Alexiou, Pere Arús, María José Aranzana

**Affiliations:** 1 IRTA (Institut de Recerca i Tecnologia Agroalimentàries), Barcelona, Spain; 2 Centre for Research in Agricultural Genomics (CRAG) CSIC-IRTA-UAB-UB, Campus UAB, Bellaterra, Barcelona, Spain

**Keywords:** Natural variation in plants, Plant breeding, Plant genetics

## Abstract

In peach, the flat phenotype is caused by a partially dominant allele in heterozygosis (*Ss*), fruits from homozygous trees (*SS*) abort a few weeks after fruit setting. Previous research has identified a SSR marker (UDP98–412) highly associated with the trait, found suitable for marker assisted selection (MAS). Here we report a ∼10 Kb deletion affecting the gene *PRUPE.6G281100*, 400 Kb upstream of UDP98-412, co-segregating with the trait. This gene is a leucine-rich repeat receptor-like kinase (LRR-RLK) orthologous to the *Brassinosteroid insensitive 1-associated receptor kinase 1* (*BAK1*) group. PCR markers suitable for MAS confirmed its strong association with the trait in a collection of 246 cultivars. They were used to evaluate the DNA from a round fruit derived from a somatic mutation of the flat variety ‘UFO-4’, revealing that the mutation affected the flat associated allele (*S*). Protein BLAST alignment identified significant hits with genes involved in different biological processes. Best protein hit occurred with *AtRLP12*, which may functionally complement *CLAVATA2*, a key regulator that controls the stem cell population size. RT-PCR analysis revealed the absence of transcription of the partially deleted allele. The data support *PRUPE.6G281100* as a candidate gene for flat shape in peach.

## Introduction

Fruits are the edible part of many cultivated species and their study is one of the major topics in plant research. Peach (*Prunus persica* (L.) Batsch) is one of the most economically important fruit species in temperate regions. The fruits are drupes which develop from a single carpel. The calyx and the stamen of the flowers fuse into the hypanthium tissue forming a cuplike structure around the ovary. All peach tissues come from the ovary; the outer skin is the exocarp, the mesocarp the edible flesh and the pit the endocarp. Most peach cultivars are round or oval shaped, although commercial interest in flat shape fruits is increasing fast. Nowadays, just in Spain 11,700 ha are cultivated in one year with flat peaches, producing 215,260 tons^1^.

While little is known about the genetic mechanisms regulating fruit morphogenesis in fruit trees, many genetic studies have aimed to unravel such process in the model species Arabidopsis. In this species leucine-rich receptor like kinases like ERECTA and CLAVATA-1, show functional implications in the maintenance, size and shape meristem^2, 3^. In particular, ERECTA regulates organ shape and flower architecture, showing the loss-of-function *erecta* mutants compact inflorescences, short pedicels and round flowers^3, 4^.

Among cultivated species, fruit shape has been most studied in tomato. The fruits are berries which develop from the ovary after fertilization of the ovules. The wall of the ovary develops into the pericarp and encloses the placenta and seeds. Four genes controlling tomato fruit shape have been cloned: SUN^5^, *OVATE* ^6^, *LOCULE NUMBER* and *FASCIATED* ^7, 8^. In addition several loci which regulate fruit shape have been identified including two suppressor elements of the ovate mutation (*Sov1* and *Sov2*)^9^. One is the mutant *Self1*, producing fruit elongation by increasing cell layers in the ovary^10^, and the other is QTL fs8.1 which also controls fruit elongation^11^. *SUN*, *OVATE*, and *fs8.1* act together in additive manner to control fruit shape producing longer fruits. In cucumber, a homolog of the tomato *SUN* gene (*CsSUM*) is a candidate for round fruit shape^12^.

Flat peaches originated in South China, where they are known as “pentao” from the original Chinese “Pan Tao”. In the mid-1800s several Chinese flat varieties were introduced into USA breeding programs as carriers of characters such as low chilling^13^, but they were popular for a brief period of time. It is believed that the first flat peach variety, bred by Starks Nursery in 1985, was ‘Saturn’ and later, in the 1990s, its cultivation became more widespread^14^.

The flat shape of the peach fruit is determined in the early stages of flower development by a single gene *S/s* (for saucer-shaped) mapped in the distal part of chromosome 6^15^. Fruits from individuals with the *ss* genotype are round, those heterozygous for the flat allele (*Ss*) are flat, and fruits from homozygous *SS* plants abort several weeks after anthesis. Although the hypothesis of a single gene explains the phenotypes observed, abortion of homozygous *SS* plants also suggests two dominant closely linked genes (*S*/*s* and *Af*/*af*) in repulsion^16^. Up to now, several markers have been identified around the *S* locus, by analyzing both mapping progenies and germplasm^17–19^. One of the markers, the SSR UDP98-412 has been reported to be tightly linked to the *S* locus and works efficiently in marker assisted selection (MAS)^18^.

Horn *et al*.^20^ mapped ESTs of 3,842 candidate genes for fruit quality in the *Prunus* reference map, but no candidate genes were identified for fruit shape. Recently, a *PpCAD1* gene (*Prupe.6G292200*, alias *ppa003772m* peach genome v.1) has been reported^19^ as candidate for the trait based on a GWAS analysis.

Here we propose a new candidate gene, *PRUPE.6G281100* (a *LRR-RLK*), whose non-functional allele is observed in heterozygosis in flat varieties and in homozygosis in aborting varieties. Its homology with genes involved in organ shape in other species makes this gene a good candidate for this trait. We also analysed a somatic mutant of a flat variety that reverted to round shape. The analysis of *PRUPE.6G281100* in the DNA from fruit tissues of the flat variety and its round mutant revealed that the non-functional allele of *PRUPE.6G281100* suffered a mutation, reinforcing the hypothesis of its role in determining peach fruit shape.

## Results

### Search for polymorphisms associated to the flat shape trait

To find DNA polymorphisms associated with the flat trait in peach, we explored a 30.2 kb region flanking the SSR UDP98-412, previously reported to be tightly linked to this trait^18^. We designed 14 primer pairs to amplify and sequence fragments of 350–680 bp in this region, in a small set of three flat and three round peaches. No polymorphisms (SNPs or INDELs) were observed in the DNA fragments amplified by these primers.

**Figure 1:**
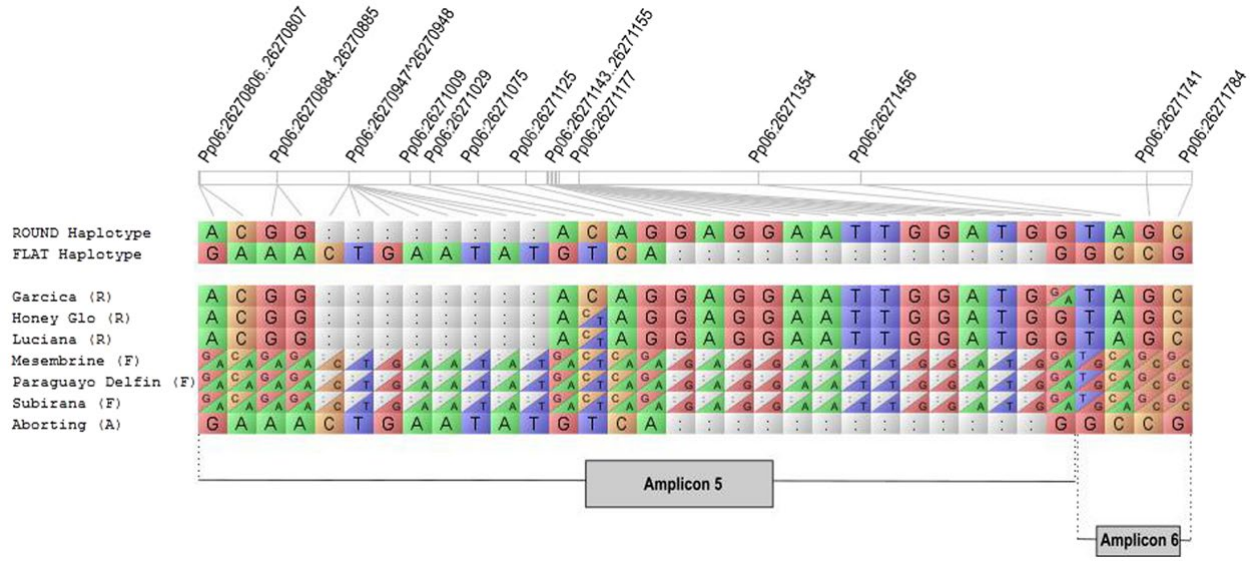
Round and flat associated haplotypes in round (R), flat (F) and aborting (A) peaches. Colon represents the deletion of a nucleotide. The haplotypes consist of 13 SNPs and two INDELS, in Amplicon5 and Amplicon6 (Pp06: 26,270,679.26,271,829) (see Supplementary Table S1).

The SNPs closest to UDP98-412 annotated in the peach genome database occur 363.5 kb upstream of this marker, with 20 SNPs in a 26.5 kb region (Pp06: 26,254,140.26,254,809). All these SNPs were located in the coding regions of five annotated transcripts. By sequencing nine amplicons of these transcripts we confirmed these 20, plus 10 additional, SNPs in the same set of six flat and round peaches. Thirteen out of the 30 SNPs were associated with the flat phenotype in the small panel of cultivars. All 13 SNPs occurred in a total of 1,150 bp of two partially overlapping amplicons, nine in Amplicon5 and four in Amplicon6 (Supplementary Table S1). In addition, we detected an insertion/deletion (INDEL) polymorphism in Amplicon5 in heterozygosis in flat varieties. To confirm the association of the SNPs and the INDEL with the phenotype we sequenced Amplicon5 and Amplicon6 in 112 varieties (65 round, 47 flat) and three aborting phenotypes (Aborting02, Aborting08 and Aborting17) from the ‘UFO3’ × ‘SweetCap’ progeny. All round varieties were homozygous for the reference allele in 11 out of the previous 13 SNPs and the flat ones heterozygous, while the aborting seedlings where homozygous for the alternative allele, in agreement with the genetics of the trait (Supplementary Table S2). Alignment of the round and aborting sequences of Amplicon5 against the reference genome gave two INDEL variants in the aborting sequence: an 8 bp insertion and, a few bases downstream, a 13 bp deletion. Forward and reverse sequences of Amplicon 5 in flat varieties revealed that they contained both INDELs in heterozygosis. By cloning and sequencing the PCR product of one flat variety (‘UFO-8’) we confirmed that each of the two alleles were identical to round and aborting, respectively. The two haplotypes observed for Amplicon5 and Amplicon6, in homozygosis or heterozygosis in the small panel of flat and round varieties, are shown in Fig. 1.

According to the genome annotation, Amplicon5 and Amplicon6 are part of coding regions of a 2,253 bp long transcript (*PRUPE.6G281100*). Custom DNA BLAST analysis with PLAZA 3.0 gave significant alignment of *PRUPE.6G281100* with seven genes in the peach genome; four (*PRUPE.6G281000*, *PRUPE.6G281200*, *PRUPE.6G281300* and *PRUPE.6G281400*) in a region of 36.4 Kb in chromosome 6 containing *PRUPE.6G281100*; one in chromosome 7 (*PRUPE.7G088700*) and the two remaining in chromosome 8 (*PRUPE.8G054400* and *PRUPE.8G054300*).

### Variant identification and allele cloning

**Figure 2:**
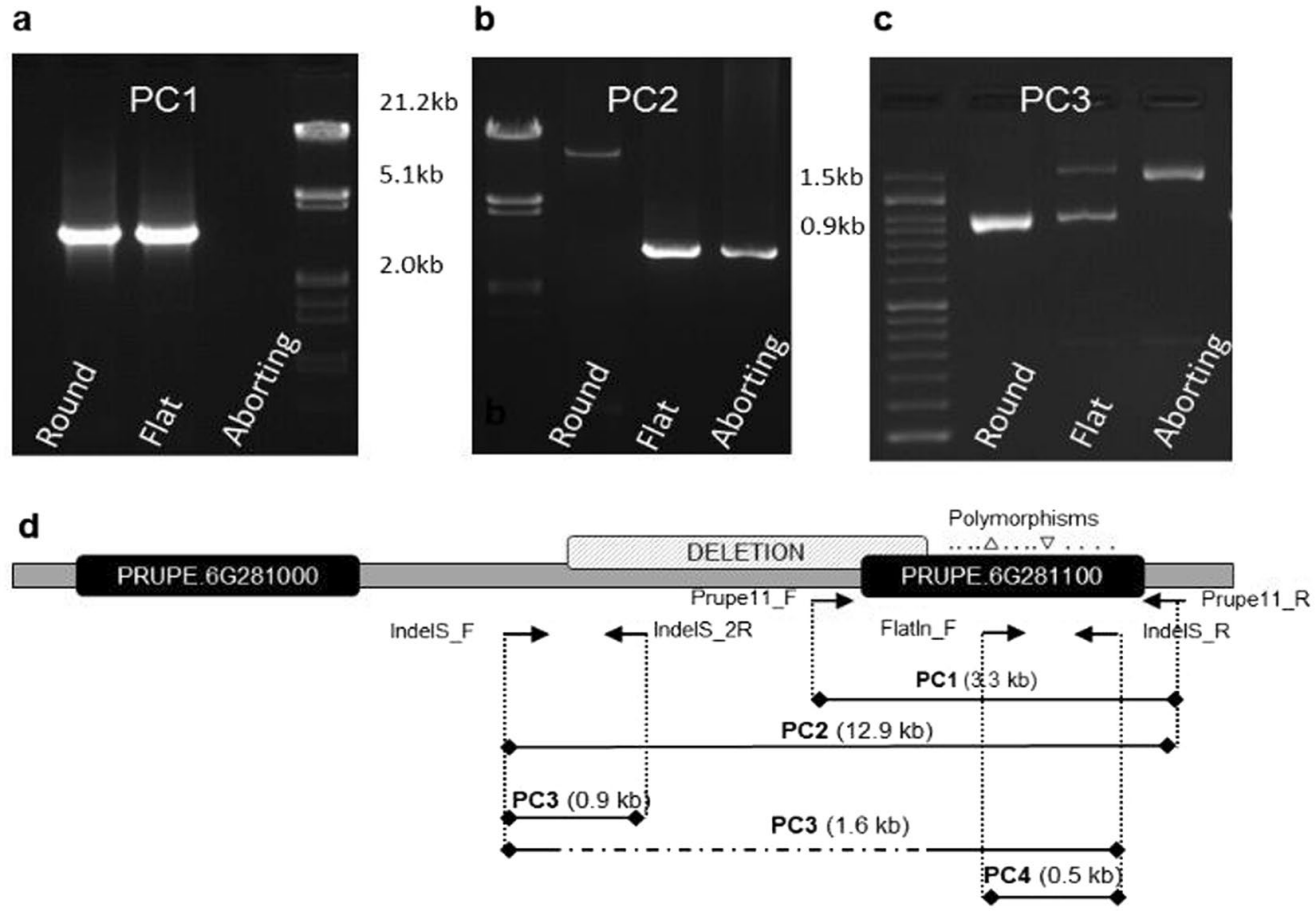
PCR bands reveal the deletion affecting the gene PRUPE.6G281100 in aborting and flat peaches. (**a**) PC1 failed to amplify the flat-associated allele. (**b**) Long-range PCR amplification with PC2 produced a fragment about 10 kb shorter in flat and aborting than in round peaches. (**c**) PCR-amplification with PC3 identified round (941pb), flat (1620/941 bp) and aborting (1620 bp) genotypes. (**d**) Diagrammatic representation of the position of the primers used to identify the polymorphisms associated with flat shape and the polymorphisms (SNPs and small INDELs) in PRUPE.6G281100, represented as dots and triangles (respectively).

To obtain the whole sequence of the flat and round associated alleles of *PRUPE.6G281100* we used primers upstream, downstream and within this gene in two round (‘Garcica’ and ‘Honey Glo’), two flat (‘Paraguayo Delfín’ and ‘Mesembrine’) and two aborting individuals. Amplification with the primer pair flanking the gene (PC1) gave a fragment with the expected size (3.3 Kb) in the round and flat DNAs, but failed with the aborting ones (Fig. 2a). The sequence of the amplified fragment in the flat varieties revealed the presence of the round allele only (lacking the two INDELs). The failed amplification of the flat-associated allele suggested a polymorphism in or near the gene.

To explore this hypothesis we amplified the samples with forward and reverse primers designed at opposite ends of the gene. Primers 10,072 bp upstream and 558 bp downstream of the gene (PC2) yielded one band of the expected size for the round sample (12,882 kb), and one about 10 kb shorter in the aborting and in the flat peaches (Fig. 2b). The full sequence of the short band revealed a fragment 2,912 nucleotides long, and consequently 9,970 bp less than that expected from the reference genome. The polymorphisms consisted in the loss of a region from 9,324 bp upstream of the start codon (Pp06: 26,260,453) to 693 bp downstream of this codon (Pp06: 26,726,336). Despite amplifying the large band in the round samples, where it occurred in homozygosis, we were not able to obtain this band in the heterozygous flat samples, where the short allele appeared to amplify preferentially. As a result, the presence of this fragment in heterozygosis was validated with a three-primer PCR assay (PC3): two (IndelS_F and IndelS_R) flanking the deletion to amplify a 1,620 bp associated to the flat phenotype and one internal to the deletion (IndelS_2 R) to produce a 941 bp band associated to the round phenotype (Fig. 2c and d).

### Variant validation with NGS

**Figure 3:**
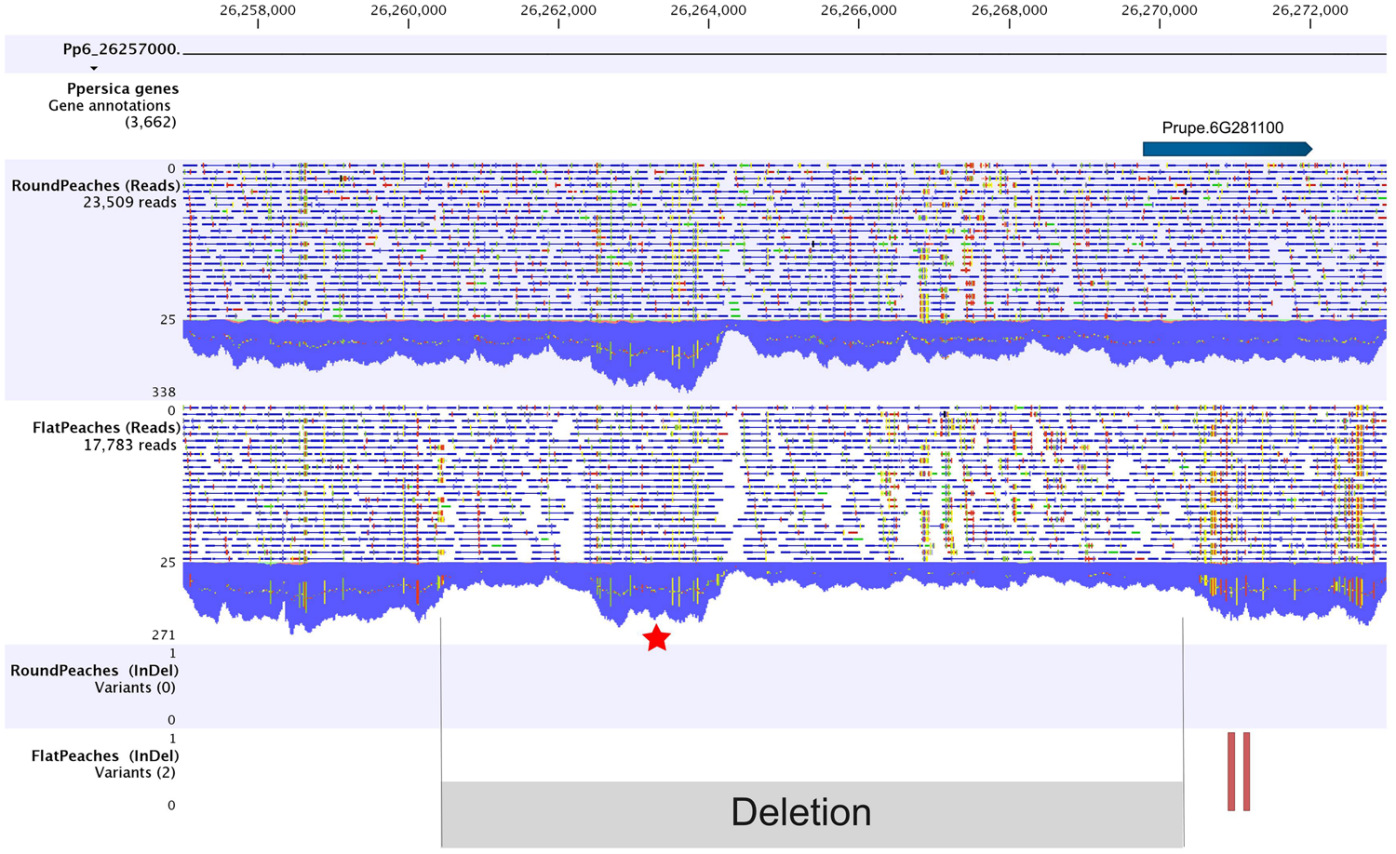
Alignment of round and flat peaches reads against Pp06:26,262,400.26,264,250 region. CLC-Workbench track display including (a) Pp06:26262400.26264250 region Prupe.6G281100, (b) bulked alignment of Illumina reads from five round and (c) five flat peaches. Blue areas represent the sequence depth at each position. The reduction in the number of reads in the flat peaches reveals the deletion in heterozygosis. An increase in the number of reads in the region Pp06:26,262,400.26,264,250 (labeled in the figure with a*) is produced by the spurious alignment (confirmed by Sanger sequencing) of a highly repetitive region. (d) The *CLC- InDels and structural variants* tool identified the two indels in the flat varieties only.

The presence of the large deletion in heterozygosis in flat varieties was also validated by resequencing five flat and five round varieties with Illumina NGS technology. The alignments corresponding to Pp06:26,257,000.26,273,000 region (16 kb) of varieties of each fruit type were bulk-analysed. With the CLC-INDELs and Structural Variants tool, the two small INDELs within the gene were identified, but not the large deletion. However this deletion was evident in the visual track of the alignment, where only flat peaches had less reads in the deleted region (Fig. 3).

### Polymorphism validation in peach germplasm and markers for seedling selection

The two small INDELs within the gene and the close to 10 kb deletion were tested with PC4 and PC3, respectively, in a panel of 177 flat, round and aborting samples (Supplementary Table S2). All genotypes matched the observed phenotype. For the two small INDELs, the size of the fragments (469 bp for the round and 464 bp for the flat-associated alleles) confirmed that the two INDEL variants were in heterozygosis in flat varieties and seedlings while the respective alleles were homozygous in round and aborting ones. Similarly, the genotype obtained with the primers flanking and within the 10 kb deletion matched the phenotype (941 bp for the round and 1,620 bp for the flat associated alleles).

### Expression analysis

**Figure 4:**
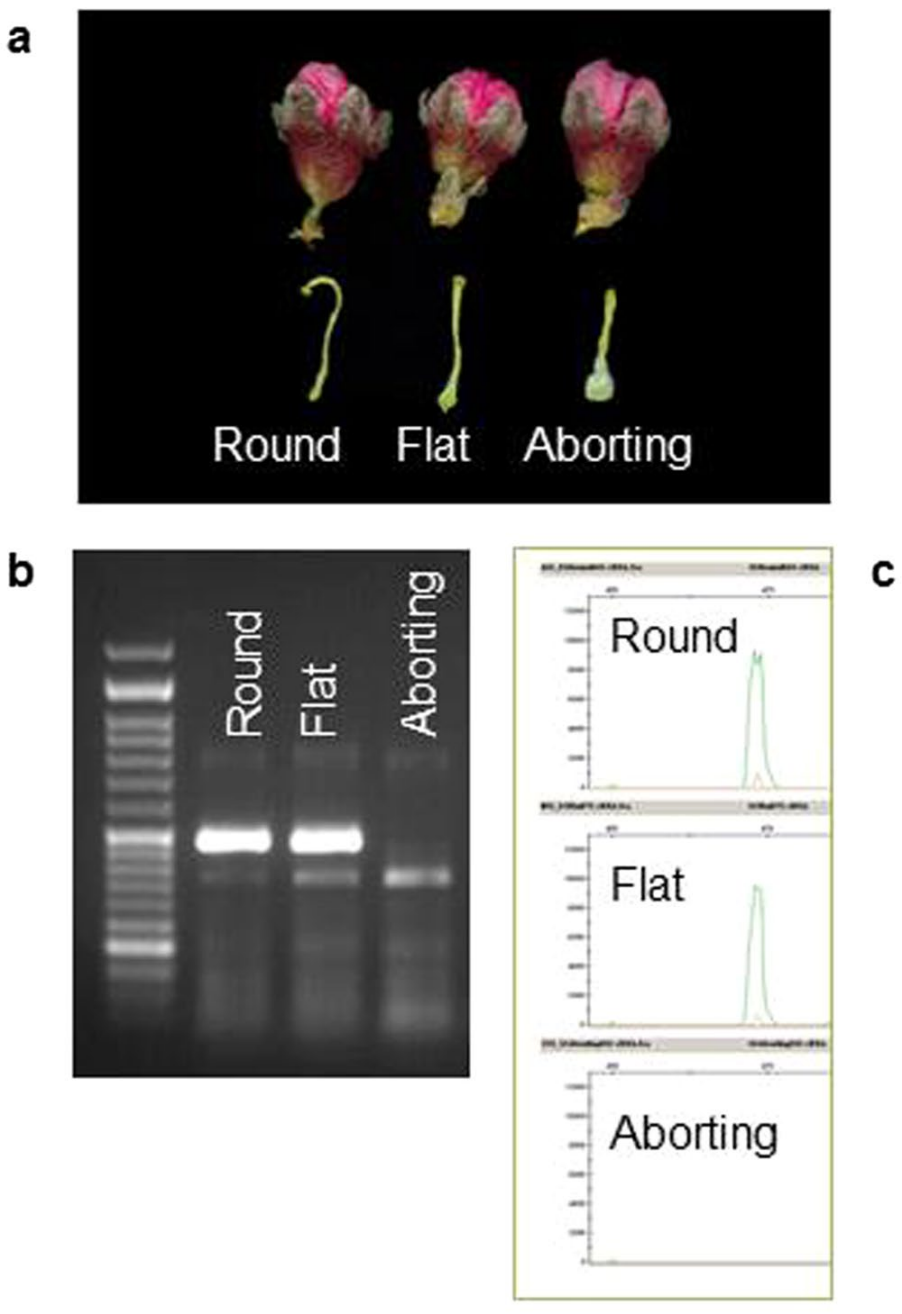
RT-PCR of RNA from round, flat and aborting pistils. (**a**) Pistil shape observed in flower buds in stage E. On RT-PCR amplification of round, flat and aborting pistils using PC4 no amplification of the flat-associated allele was visible in both (**b**) agarose and (**c**) capillary electrophoresis.

RT-PCR amplification of RNA extracted from pistils of round, flat and aborting peaches produced fragments exclusively from the round and flat samples (Fig. 4). The size of the bands and their sequence revealed that, in both cases, the fragment amplified corresponded to the round-associated allele (lacking the two small INDELs), indicating the absence of transcription of the flat-associated allele.

### Homology and functional prediction of the gene

The gene *PRUPE.6G281100* (allias *ppa025511m* in the peach genome annotation v.1) is a leucine-rich repeat kinase (PLAZA 3.0 gene family HOM03D000009, orthologue group ORTHO03D000261 described as Brassinosteroid insensitive 1-associated receptor kinase 1, *BAK1*). BLAST analysis of the translated protein against the PLAZA protein sequence database gave best alignments with 250 genes from 55 subfamilies of the gene family HOM03D00009 (Supplementary Table S3). Most of the genes (160 genes, 64%) belonged to four subfamilies (ORTHO03D000261, ORTHO03D000539, ORTHO03D001987, ORTHO03D002896). Thirty-eight of them were annotated as BAK1 (23.8%), 30 (18.8%) as leucine-rich repeat receptor-like kinase protein *FLORAL ORGAN NUMBER1* and 81 (50.6%) as leucine-rich repeat receptor–like kinase protein *THICK TASSEL DWARF1* in 25 species, including peach and other *Rosaceae* members such as *Fragaria vesca* and *Malus* x *domestica*. Within peach, these were *PRUPE.6G281000*, *PRUPE.6G281200*, *PRUPE.6G281300*, *PRUPE.6G281400*, *PRUPE.6G281500*, *PRUPE.6G288800*, *PRUPE.7G088700*, *PRUPE.8G054400* and *PRUPE.8G054300*.

Protein BLAST pairwise alignment against the SwissProt Arabidopsis database gave significant hits with LRR-RLK, involved in different biological processes. Best hit occurred with the AtRLP12 gene. This gene may functionally complement *CLAVATA2*, a key regulator that acts at the shoot apical meristem (SAM) of plants, controlling the stem cell population size^21^.

### Analysis of the polymorphism in a round somatic mutant

**Figure 5:**
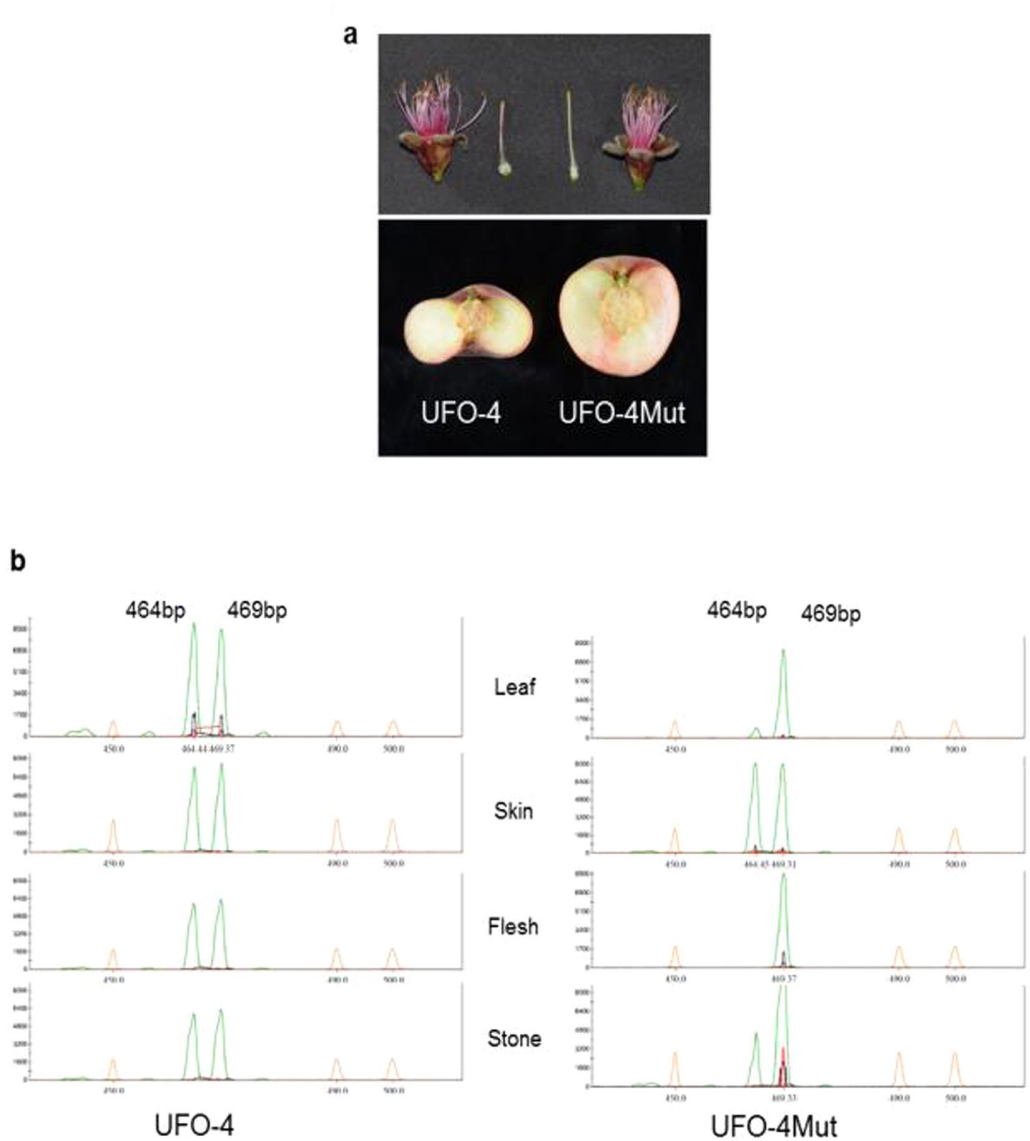
Analysis of a flat variety (‘UFO-4’) and its somatic round mutant (‘UFO-4Mut’). (**a**) Image of the flat (left) and round (right) pistils and fruits. (**b**) PCR-amplification products obtained with PC4 to detect the two small INDELs associated with the flat trait. PCR reactions were carried out with DNA extracted from leaf, as well as from skin, flesh and stone fruit tissues. The peaks show that the mutation occurred in flesh tissue (meristematic layer LII) affecting the flat associated allele.

We analyzed the polymorphisms in a round peach generated from a somatic natural mutant of the flat variety ‘UFO-4’ (Fig. 5). The analysis of genomic DNA with PC4 (with formward and reverse priers flanking the two small INDELs inside PRUPE.6G281100) showed a faint amplification of the flat allele in the mutated round cultivar compared to the strong signal observed in the original flat. Amplification of DNA extracted from skin, flesh and stone tissues revealed the absence of the flat associated allele in the flesh mutated DNA while it was present in the skin DNA, indicating that the mutation occurred in the meristematic LII. Faint amplification of the flat allele was observed in the stone DNA of the mutant, which could be due to the invasion of LIII by mutated LII cells.

As for ‘UFO-4’, amplification of ‘UFO-4Mut’ flesh DNA with the primers flanking *PRUPE.6G281100* (PC1) produced only the round-associated allele, as occurred with those designed to genotype the 10 kb deletion (PC3). These results suggest a mutation affecting the flat associated allele which could have caused the reversion of the phenotype from flat to round.

## Discussion

Here we explored the genetic variability in a region associated with the flat shape in peach and identified a candidate gene for this trait. Considering the high level of variability observed genome-wide between round and flat peaches^22^ the large extension of LD in peach^23, 24^ and the codominant mode of action of the flat and round alleles, which must be in heterozygosis in flat varieties, we expected to find a substantial level of heterozygosis in the region flanking the SSR marker associated with the trait (UDP98-412). Surprisingly none of the fragments sequenced showed polymorphisms. Thereafter we searched for the closest region to UDP98-412 with annotated SNPs in the databases. This region was 337.5 Kb upstream and contained one SNP every 521 bp, close to the density of 1 SNP every 598 bp found by ref. 25 after sequencing genes in peach varieties, but much higher than the density of 1 SNP every 1076 bp observed by ref. 26 in Chinese edible varieties.

After sequencing nine amplicons of the variable region in a panel of varieties we identified SNPs highly associated with flat, round and aborting phenotypes in two amplicons of the gene *PRUPE.6G281100*. By amplification, cloning and sequencing part of the gene, 11 SNPs and two INDELs co-segregating with the trait were identified (Fig. 1), which allowed us to design an allelic specific marker diagnostic for this trait. This marker was validated in 177 varieties from different origins, including nineteen where UDP98-412 alleles escaped association with the trait^18^. In all cases, the genotype obtained was in agreement with fruit shape phenotype, confirming that this region is closer to the *S/s* locus. In consequence, we provide here a simple marker (FlatIn_F/IndelS_F; PC4) able to amplify two fragments differing in 5 bp, that improves the performance of UDP98-412 and is more efficient for MAS. Additionally to this primer combination, we found several SNPs that can be used for the same purpose. Long-range PCR reactions detected a ~10 Kb deletion of part of the gene (693 bp from the ATG starting codon), affecting the flat-associated allele. Alignment and coverage analysis of NGS reads of five flat and five round varieties allowed visualization of the alignment of the large gap in heterozygosis (Fig. 3). This deletion was validated in the panel of varieties, with all flat varieties sharing the same haplotype and suggesting a unique origin of the flat trait in the panel evaluated. Some of these varieties (18) have been analyzed in ref. 27 with an SNP array of close to 9,000 SNPs. They were distributed along all transects of the variability observed including, the major Oriental and Occidental clusters, indicating that the varieties analyzed here covered a broad range of variability. Unlike peaches and nectarines that are separated in different clusters in Occidental materials^23^, no specific clusters including only flat peaches occur, which is consistent with the fact that, due to the heterozygous nature of flat peaches, breeding is usually by selecting in round x flat progenies, and that the flat allele, originating from a single source, may have been introgressed in a diverse array of materials.

RT-PCR (Fig. 4) and posterior band sequencing revealed the absence of transcription of the flat associated allele, indicating a loss of function of *PRUPE.6G281100*. Thereafter *PRUPE.6G281100* gene, from the orthologous group ORTHO03D000261, annotated as Brassinosteroid insensitive 1-associated receptor kinase 1 (*BAK1*) arises here as a candidate for the flat shape of peach fruits. This gene is a leucine-rich repeat receptor-like kinase (LRR-RLK), the proteins constituting ligand-receptor systems that control cell fate specification, and mediate correct cell divisions and cell-to-cell communication, allowing correct generation of tissues and organs through growth and development of both animals and plants^28^. Plant RLKs can be classified into six classes based on the structural feature of the extracellular domain. The largest class of plant RLKs is the LRR-RLKs class (700 in *Arabidopsis* and 1,400 in rice)^29^, proteins that contain leucine-rich repeats, which are tandem repeats of approximately 24 amino acids with conserved leucines involved in protein-protein interactions. Most LRR-RLKs are involved in embryonic pattern formation, which suggests a putative role of this protein in the coordination of cell proliferation during embryogenesis and during morphogenesis of embryonic cells at meristems, shaping the plant^30^. Two LRR-RLKs, *CLAVATA1* and ERECTA, show functional implications in the maintenance, size and shape of meristems^2^.

The protein showed best homology with *BAK1*, *FLORAL ORGAN NUMBER1* (*FON1*) and *THICK TASSEL DWARF1* (*TD1*) genes. *BAK1* is involved in brassinosteroid (BR) signal transduction, forming heterodimers with *BRASSINOSTEORID INSENSITIVE* (*BRII*) modulating growth and development, including cell expansion and reproductive development in species such as Arabidopsis and rice^31, 32^. The *FON1* gene encodes a receptor-like kinase protein (orthologous to *Arabidopsis CLAVATA1*) that regulates the size of the floral meristem, causing enlargement in *Oryza sativa* ^33^. Similarly, *TD1* encodes a maize orthologous to *CLAVATA1* in Arabidopsis, modulating meristem size during inflorescence and flower development and involved in the regulation of meristem structural organization^34^. We can therefore hypothesize that the LRR-kinase protein encoded by *PRUPE.6G281100* is involved in a cell signaling pathway, during flower development, that ensures a final round shape of the ovary, and consequently of the fruit. While the loss of function of this gene in homozygosis produces unviable fruits, in heterozygosis the allele produces flat fruits. This behavior resembles the mechanism of a haploinsufficient locus. Loss-of-function alleles at haploinsufficient loci are typically dominant because the level of gene function in a heterozygote is below the threshold for producing a wild-type phenotype, and homozygotes typically exhibit more severe phenotypes, including early lethality. The most common explanation is that these loci are involved in cellular processes sensitive to dosage effects and changes in protein concentration^35^.

We found several homologous genes in the peach genome, which, as suggested for Arabidopsis^31^ might be functionally redundant. Given that most of its homologues clustered in the same genome region, the large LD in peach and the unusually high variability detected in the haplotype, we need to explore other possible functional polymorphisms in the region acting alone or in combination.

Our candidate gene differs from that suggested by ref. 19, identified through a GWAS approach. These authors found associated SNPs in the fifth intron of a CONSTITUTIVELY ACTIVATED CELL DEATH GENES (*CAD1)* homologous gene, which negatively controls the salicylic acid (SA) mediated pathway of programmed cell death in plant immunity. This gene is 650 Kb downstream from our candidate gene. Flat varieties contained the polymorphism in either homozygosis (A/A) or heterozygosis (A/T), while round varieties were always homozygous T/T, indicating that these genotypes do not fully correspond with the inheritance of the trait (A/A genotypes should not produce viable fruits). To our knowledge^19^, study is the first to report a putative role of *CAD1* genes in organ shape and development and its high association with the trait could be due to the large LD extension in peach. However we cannot discard possible involvement of both genes in the trait.

Gene function is usually validated by genetic transformation or by the screening of mutants. The main obstacle in validating candidate genes in peach through genetic transformation is the regeneration of transformed plantlets. Although the transformation and regeneration of stable transgenic plantlets in peach has been reported^36, 37^, this is not yet a well resolved method.

Alternatively, the study of somatic mutants in woody plants, and in particular in peach, has been successfully used to investigate causal genes^38, 39^. These mutations often occur in only one histogenic layer, so are chimeric and most are not sexually transmitted. In peach, the histogenic layer LI gives rise to epidermal tissues, LII to subepidermal tissues, and the male and female sporogenous tissues, and LIII to the remainder of the shoots. In fruits, LI produces the skin, LII the flesh and LIII the stone.

Here we investigated the *PRUPE.6G281100* gene in a chimeric natural mutation occurring in the meristematic LII (producing the fruit flesh tissue), which reverted from the flat to the round phenotype (Fig. 5). Although we did not obtain the sequence of the mutated flat allele, the analysis of flesh DNA with allele specific primers to amplify part or the gene revealed a new structural mutation affecting the flat allele, while the skin DNA shows the intact flat and round-associated alleles. One hypothesis for the gain of function compatible with the haploinsuficiency mechanism is the recombination of the mutant flat allele with others of the LRR-Kinase genes present in the candidate gene region. As demonstrated by ref. 40, chimeric kinase receptors made in the lab can produce new functional receptors. In fact, sequence divergence, genetic recombination, duplication events and selective forces have been proven to be the main forces for the continuous RLK gene expansion^41^. Alternatively, chromosome replacement of the ‘flat’ region by the homologous ‘round’ region is also a plausible hypothesis. In grape, this type of molecular mechanism produced the mutant Pinot blanc from Pinot gris^42^. Cloning the new mutated allele will provide information of the gene mechanism.

## Methods

### Plant material

In total we studied 249 peach individuals, classified as round, flat or aborting (in those cases where either all or most of fruit set stopped within a few weeks after pollination). Of these, 177 corresponded to peach cultivars (110 round and 67 flat; see Supplementary Table S2). Seventy-one were F1 seedlings from the cross between the two flat peaches ‘UFO-3’ × ‘Sweet cap’ (with round (14), flat (40) and aborting (17) phenotypes) and three were round, flat and aborting seedlings (P07F202A065, P07F202A071 and P07F202A056, respectively) from ‘ASF08.81’ open pollination. In addition, we included a flat peach variety (‘UFO-4’) and its round somatic mutant (‘UFO-4Mut’). Buds of the branch containing the mutation were grafted and maintained at the greenhouse facilities of IRTA in Torre Marimon (Barcelona).

### DNA and RNA extraction

DNA from all materials used was extracted from young leaves using the Doyle and Doyle method^43^. For ‘UFO-4’ and ‘UFO-4Mut’ DNA was extracted from leaves, flesh fruit, skin and stone using the DNAsy Qiagen kit (Qiagen, Hilden, Germany). Branches with flowers in Baggiolini stage E (not expanded petals) from round (P07F202A065), flat (P07F202A071) and aborting (P07F202A056) peaches (all progenies from ‘ASF08.81’ open pollination) were cut in the field, and pistils collected and frozen in liquid nitrogen and conserved at −80 °C prior to total RNA extraction using the RNeasy® Plant Mini Kit (Qiagene) following the manufacturer’s protocol. RNA integrity was confirmed by 1% agarose gel electrophoresis.

### DNA genotyping to identify new polymorphisms associated with the trait

All samples were genotyped with the SSR marker UDP98-412 (Pp06: 26,617,638.26,618,013) using the PCR and electrophoresis conditions described in Picañol *et al*.^18^.

Using the peach genome sequence v.2^44^ we designed 23 primer pairs to amplify fragments of 200–700 bp in a 388.6 kb region (Pp06: 26,254,140.26,642,759) (Supplementary Table S1). Fourteen of them (primers UDP98-412(−17 K) to UDP98-412(+25 K)) were designed covering a 42.8 Kb region (Pp06: 26,599,970.26,642,759) flanking UDP98-412 and the nine remaining (primers Amplicon 1 to Amplicon 9) in a region spanning 26.6 Kb (Pp06: 26,254,140.26,280,026) 363.5 Kb upstream. This region was the closest to UDP98-412, where SNPs of the 9 K peach chip^45^ were identified. Primers were designed using Primer3 software^46^ avoiding amplification of SSR regions.

Primers were first tested in six varieties, three with flat (‘Mesembrine’, ‘Paraguayo delfín’ and ‘Subirana’) and three with round fruits (‘Garcica’, ‘HoneyGlo’ and ‘Luciana’). PCR products with a single band were purified with Exosap-it (GE HealthcareLife Science) in a single pipetting step and used as template for sequencing using the BigDye^TM^Terminator Cycle Sequencing Kit (Applied Biosystems, Foster City, CA, USA) and forward primers. The sequencing reaction profile included an initial denaturation at 96 °C for 1 m, followed by 25 cycles of 96 °C for 10 s, 50 °C for 6 s, and 60 °C for 4 min; the sequences obtained with an ABI Prism 3130 × l DNA Analyzer (Applied Biosystems, Foster City, California, CA, USA) were visualized and manually edited with Sequencher 5.0 software (Gene Codes Corporation; Ann Arbor, MI, USA). Fragment ends were trimmed to remove low-quality sequence. Haplotypes were graphically represented with Flapjack software^47^.

### Cloning of PCR fragments

For one flat variety (‘UFO-8’) PCR products were cloned into the pGEM T-easy vector (Promega) following the manufacture instructions. *Escherichia coli* DH5alpha electro competent cells (Invitrogen) were transformed with the ligated plasmid by electroporation in the Gene PulserXcel electroporation system (BIORAD), with a capacitance 25 μF, resistance of 200 ohm and a voltage of 1,8 kv. Transformed cells were shaken horizontally at 250 rpm and 37 °C for 1.5 h in 1 ml liquid Luria-Bertani (LB) medium. Fifty microliters of transformed cell solution was then pipetted onto 10 cm LB agar plates containing 50 µg/ml ampicillin, 80 µg/ml X-gal and 0.5 mM isopropyl-β-D-1-thiogalactopyranoside (IPTG). Positive colonies were picked from the LB plates as template DNA for colony PCR. Colonies were screened by PCR following the conditions described above. Those carrying the desirable allele were grown in 5 mL of LB liquid broth containing 50 µg/ml of carbenicillin with overnight incubation at 37 °C in a shaking oven at 250 rpm. Bacterial culture pellets were obtained by centrifugation at 3000 rpm for 10 min. Plasmids were extracted from bacterial cells using a QIAprep miniprep spin-kit (Qiagen) according to the manufacturer’s protocol, then resuspended in 50 μl of sterile water and 4 µl of each extract were sequenced with the vector specific primers, either T7 or SPS6, following the sequencing protocol previously described.

### Sequencing PRUPE.6G281100

#### Round shape-associated allele

Using the peach genome sequence as reference, we designed seven primer pairs (Supplementary Table S4) flanking and within *PRUPE.6G281100* (Pp06:26,269,777.26,272,029) to obtain the full sequence of the gene. Primers were designed to amplify single fragments, avoiding amplification of duplicated regions. Amplification and sequencing reactions were as described above.

#### Flat shape-associated allele

The forward primer Prupe.6G281100(−10K)_F was designed 10,072 bp upstream *PRUPE.6G281100* and was combined with the reverse primer Prupe.6G281100_3PrimF (primer combination 2 (PC2) in Supplementary Table S4), 558 bp downstream of the gene, to amplify fragments with an expected size of 12.9 Kb. For long-range PCR, LongAmp® *Taq* Polymerase (New England BioLabs ®_INC_) was used. Each reaction contained 1x LongAmp reaction buffer, 0.3 mM dNTP mix, 0.8 µM each primer, 5% DMSO, 5 units of polymerase, 40 ng of template DNA, and sterile Milli-Q water to a final volume of 25 µl. The following PCR protocol was performed on a S-1000^TM^ Thermal Cycler (Bio-Rad Laboratories, Inc.; Hercules, California, USA): 95 °C for 5 min; 35 cycles of 95 °C (30 sec), 60 °C (30 sec) and 65 °C (17 min); followed by a final step at 65 °C for 10 min. All PCR amplicons were checked on 1% agarose gel in TAE buffer. Ethidium bromide staining was used for band visualization.

The PCR bands were purified with the High Pure PCR product purification kit (Roche Diagnostic, Basel, Switzerland). Thirty nanograms of purified product were used as template to obtain the whole sequence of the amplicons in four sequencing reactions using the primers Prupe.6G281100(−10K)_F, Prupe.6G281100_4 R, Prupe.6G281100_5 R and Prupe.6G281100_3PrimF (Supplementary Table S4).

### Variant validation with NGS

To validate the large variant alignment we re-sequenced, with Illumina technology (27×), five flat (‘Flatmoon’, ‘Cakereine’, Blanvio-10’, ‘Subirana’, ‘UFO-4’) and five round (‘Nectalady’, ‘Armking’, ‘Belbinette’, ‘Nectaross’, ‘Tifany’) varieties. High quality DNA of each sample was delivered to the CNAG (Centre Nacional d’Anàlisi Genòmica, Barcelona) for library preparation and 2 × 100 pb paired-end sequencing using illumina HiSeq. 2000 sequencer. Adapter removal and quality-based trimming of the raw resequencing data was with Trimmomatic version 0.36^48^. FastQC (http://www.bioinformatics.babraham.ac.uk/projects/fastqc) was used for read quality control before and after trimming. High quality reads were mapped to the peach genome version 2.0 using BWA^49^ and the resulting alignment files were sorted and filtered by discarding multi-mapped reads and annotating PCR duplicates. Reads mapping to the Pp06:26,257,000.26,273,000 region were extracted from the alignment files and bulk aligned against the reference peach genome v2 using CLC-genomics workbench 8.5.1 (https://www.qiagenbioinformatics.com/). CLC-InDels and Structural Variants analysis tools were run separately in the flat and round peach alignments. Genes annotated in v.2 were downloaded from the GDR database^50^ and included in the alignment track for visualization. Reads are available at the European Nucleotide Archive under the accession number ENA: PRJEB21538.

### Design of markers for genotyping

To validate the polymorphisms in germplasm and progenies, we designed primers to amplify a small INDEL within the candidate gene as well as the large deletion upstream from the candidate gene. Thus primer pairs FlatIn_F and IndelS_R (both inside the gene; PC4 in Supplementary Table S4) yielded product sizes of 464 bp and 469 bp for the flat and round alleles, respectively. PCR conditions, fragment separation and analysis in the ABI Prism 3130 × l DNA Analyzer were as previously described for the SSR marker.

A three-primer combination (PC3 in Supplementary Table S4), consisting of two primers flanking the deletion (one forward and one reverse) and an inner reverse primer (IndelS_F + IndelS_2 R + IndelS_R), was designed to genotype the large deletion identified in this region; IndelS_F and IndelS_R (flanking the deletion) amplified a 1,620 bp fragment associated to the flat phenotype, while IndelS_2 R (within the deletion) in combination with IndelS_F produced a 941 bp band associated to the round phenotype. PCR was carried out in a 10 µl reaction containing 20 ng of DNA, 1x PCR buffer, 1.5 mM MgCl_2_, 200 µM of each dNTP, 0.2 µM of each primer and 1 U of BIOTAQ (Biolab). The following PCR protocol was used in a S-1000^TM^ Thermal Cycler (Bio-Rad Laboratories, Inc.; Hercules, California, USA): 95 °C for 2 min; 35 cycles of 94 °C (30 sec), 55 °C (30 sec), 72 °C (60 sec); followed by a final step at 65 °C for 10 min. All PCR amplicons were checked on 1% agarose gel in TAE buffer. Ethidium bromide staining was used for band visualization.

### RT-PCR analysis

RNA was reverse-transcribed to cDNA using the reverse primer IndelS_R (see Supplementary Table S4). For this, 1 µl of RNA was hybridized with 2 µl of primer in a total volume of 13 µl. After 10 min incubation at 70 °C and 5 min cooling on ice, cDNA was obtained using PrimeScript RT-PCR kit (Takara). PCR was conducted with PC4 following the protocol described above. The forward primer was florescent labeled to check the size of the fragment in the ABI Prism 3130 × l DNA Analyser.

### Gene homology and functional prediction

Functional annotation and orthologues for *the PRUPE.6G281100* gene were determined using Dicots PLAZA 3.0. (http://bioinformatics.psb.ugent.be/plaza/versions/plaza_v3_dicots/^51^). Custom DNA BLAST (blastn program) against PLAZA Transcript Sequences database were used for similarity searches, filtering for low complexity and using the BLOSUM62 score matrix.

For the protein sequence of *PRUPE.6G281100*, associated with the round allele, the DNA sequence was entered in the Translate tool of the ExPASy Bioinformatics Resource Portal (http://web.expasy.org/translate/). Similarity searches were performed on the NCBI web page (www.ncbi.nlm.nih.gov) against the nr (non-redundant) collection of sequences in GenBank and the UniProtKB/SwissProt databases, using the blastp and the Position-Specific iterated BLAST algorithm^52^. The quality of the pairwise sequence alignment was evaluated in a BLOSUM62 protein substitution matrix allowing a gap existence value of 11 and an extension value of 1.

## Electronic supplementary material

Supplementary Tables S1 to S4
