# Author Correction: A deletion affecting an LRR-RLK gene co-segregates with the fruit flat shape trait in peach

**DOI:** 10.1038/s41598-022-09334-2

**Published:** 2022-03-30

**Authors:** Elena López-Girona, Yu Zhang, Iban Eduardo, José Ramón Hernández Mora, Konstantinos G. Alexiou, Pere Arús, María José Aranzana

**Affiliations:** 1 IRTA (Institut de Recerca i Tecnologia Agroalimentàries), Barcelona, Spain; 2 Centre for Research in Agricultural Genomics (CRAG) CSIC-IRTA-UAB-UB, Campus UAB, Bellaterra, Barcelona, Spain

Correction to: *Scientific Reports* 10.1038/s41598-017-07022-0, published online 27 July 2017

The Acknowledgements section in the original version of this Article is incomplete.

“We thank Cristian Fontich (IRTA) and Jesús García Brunton (IMIDA) for maintaining and providing peach material. This work received financial support from the Spanish Ministry of Economy and Competitiveness, through the “Severo Ochoa Programme for Centres of Excellence in R&D” 2016–2019 (SEV-2015-0533)” and through the project AGL2015-68329-R, and from the CERCA Programme/Generalitat de Catalunya.”

should read:

“We thank Cristian Fontich (IRTA) and Jesús García Brunton (IMIDA) for maintaining and providing peach material. This work received financial support from the Spanish Ministry of Economy and Competitiveness, through the “Severo Ochoa Programme for Centres of Excellence in R&D” 2016–2019 (SEV-2015-0533)” and through the project AGL2015-68329-R funded by MINECO and FEDER, and from the CERCA Programme/Generalitat de Catalunya.”

